# Increased Catalase Activity and Maintenance of Photosystem II Distinguishes High-Yield Mutants From Low-Yield Mutants of Rice var. Nagina22 Under Low-Phosphorus Stress

**DOI:** 10.3389/fpls.2018.01543

**Published:** 2018-11-19

**Authors:** Yugandhar Poli, Veronica Nallamothu, Divya Balakrishnan, Palakurthi Ramesh, Subrahmanyam Desiraju, Satendra Kumar Mangrauthia, Sitapathi Rao Voleti, Sarla Neelamraju

**Affiliations:** ^1^ICAR-Indian Institute of Rice Research, Hyderabad, India; ^2^Department of Biotechnology, Yogi Vemana University, Kadapa, India

**Keywords:** upland rice variety, EMS-induced mutants, low P condition, antioxidant enzymes, *F*_v_/*F*_m_ test

## Abstract

An upland rice variety, Nagina22 (N22) and its 137 ethyl methanesulfonate (EMS)-induced mutants, along with a sensitive variety, Jaya, was screened both in low phosphorus (P) field (Olsen P 1.8) and in normal field (Olsen P 24) during dry season. Based on the grain yield (YLD) of plants in normal field and plants in low P field, 27 gain of function (high-YLD represented as hy) and 9 loss of function (low-YLD represented as ly) mutants were selected and compared with N22 for physiological and genotyping studies. In low P field, hy mutants showed higher P concentration in roots, leaves, grains, and in the whole plant than in ly mutants at harvest. In low P conditions, *F*_v_/*F*_m_ and q_N_ were 24% higher in hy mutants than in ly mutants. In comparison with ly mutants, the superoxide dismutase (SOD) activity in the roots and leaves of hy mutants in low P fields was 9% and 41% higher at the vegetative stage, respectively, but 51% and 14% lower in the roots and leaves at the reproductive stage, respectively. However, in comparison with ly mutants, the catalase (CAT) activity in the roots and leaves of hy mutants in low P fields was 35% higher at the vegetative stage and 15% and 17% higher at the reproductive stage, respectively. Similarly, hy mutants in low P field showed 20% and 80% higher peroxidase (POD) activity in the roots and leaves at the vegetative stage, respectively, but showed 14% and 16% lower POD activity at the reproductive stage in the roots and leaves, respectively. Marker trait association analysis using 48 simple sequence repeat (SSR) markers and 10 *Pup1* gene markers showed that RM3648 and RM451 in chromosome 4 were significantly associated with grain YLD, tiller number (TN), SOD, and POD activities in both the roots and leaves in low P conditions only. Similarly, RM3334 and RM6300 in chromosome 5 were associated with CAT activity in leaves in low P conditions. Notably, grain YLD was positively and significantly correlated with CAT activity in the roots and shoots, *F*_v_/*F*_m_ and q_N_ in low P conditions, and the shoots’ P concentration and q_N_ in normal conditions. Furthermore, CAT activity in shoots was positively and significantly correlated with TN in both low P and normal conditions. Thus, chromosomal regions and physiological traits that have a role in imparting tolerance to low P in the field were identified.

## Introduction

Factors influencing crop growth and productivity, nutrients being the most important, are applied in the form of fertilizers. Among these nutrients, phosphorus (P), secondary to nitrogen (N), plays an essential role in plant growth. Phosphorus fixation is a common problem in the soils of rice fields (Krishnamurthy et al., 2010; Panigrahy et al., 2014; Vandamme et al., 2016; Yugandhar et al., 2018a,b). The applied P is not completely available to plants because of slow diffusion rates and processes such as fixation in alkaline soils with Ca and fixation in acidic soils with Fe and Al. For the farming community, P fertilizers are costly and difficult to access, which cause major concerns for rice cultivation in India and other rice growing countries. Phosphorus fertilizer production is largely dependent on rock phosphate, a nonrenewable resource, and this finite source of P is under the threat of exhaustion.

Low P availability reduces plant height (PH), tiller number (TN), and yield (YLD) attributes such as the number of panicles, grains per panicle, filled grains per panicle, and higher spikelet sterility, thus limiting plant productivity (Veronica et al., 2017). Increase of root acid phosphatase is one of the most common responses to low P conditions, which help plants to hydrolyze organic P into inorganic P and enhance the availability of P to plants (Mehra et al., 2017; Pandey et al., 2017). For this reason, there is a need to develop genotypes that can produce an acceptable grain YLD under low P conditions. In India, the availability of P in most of the cultivated soils is becoming either low or extremely poor (Sanyal et al., 2015), particularly in upland soils.

Phosphate deprivation lowers the leaf photosynthetic oxygen evolution, photosystem II quantum efficiency, and electron transport (Veronica et al., 2017). Reactive oxygen species (ROS) act as signaling molecules under normal physiological conditions and play a vital role in signal transduction. Exposure of plants to low P conditions triggers an increased generation of reactive oxygen intermediates (ROI) or ROS such as O^2-^, H_2_O_2_, and OH^-^ radicals, which are cytotoxic and disrupt the normal metabolism through oxidative damage of lipids, proteins, and nucleic acids, leading to oxidative stress. To manage these ROS outbreaks, plants have evolved intricate antioxidant defense systems, consisting of detoxifying or ROS scavenging enzymatic antioxidants, namely superoxide dismutase (SOD), peroxidase (POD), catalase (CAT), glutathione reductase (GR), and non-enzymatic antioxidants, namely glutathione, ascorbic acid, carotenoids, α-tocopherol, and flavonoids. In rice root tissues, the activities of these three scavenging enzymes (SOD, POD, and CAT) are affected by phosphate deficiency (Juszczuk et al., 2001). Decreased photosynthetic efficiency was reported in low P field conditions, in crops such as rice (Veronica et al., 2017), wheat (Rodríguez et al., 1998), and soybean (Lauer et al., 1989). Veronica et al. (2017) have screened four rice genotypes for antioxidant enzyme activities in 0, 15, 30, 45,and 60 kg/ha of applied P in soil and found that higher CAT activity in the rice cultivar Swarna at the flowering stage led to higher grain YLD in low P conditions. Hussain et al. (2016) reported that P deprivation led to a significant increase in POD and CAT activities in 18-day-old rice hydroponically grown seedlings.

Nagina22, a deep-rooted upland rice variety, tolerant to various biotic and abiotic stresses, was subjected to ethyl methane sulfonate (EMS)-induced mutations. For characterization, 85,000 EMS-induced mutants were generated and maintained under a multi-institutional program (Mithra et al., 2016). Mutants for abiotic stresses such as heat, drought, low-P tolerance, and herbicide resistance were identified (Poli et al., 2013, 2017a,b; Mohapatra et al., 2014; Panigrahy et al., 2014; Lima et al., 2015; Talla et al., 2016; Shoba et al., 2017; Yugandhar et al., 2018a).

In this study, we evaluated different physiological and biochemical traits, including the activity of antioxidant enzymes in the roots and leaves as well as fluorescence parameters in flag leaves. The 27 gain of function (high-YLD represented as hy) and 9 loss of function (low-YLD represented as ly) mutants were compared with N22 and Jaya under normal as well as low P conditions to identify traits that can distinguish hy and ly mutants in low P conditions. In order to establish marker trait associations, mutants were genotyped using simple sequence repeat (SSR) and *Pup1* gene linked markers.

## Materials and Methods

Field experiments were carried out at ICAR-IIRR (Indian Institute of Rice Research) in 2013. Rice [*Oryza sativa* L. sub group *aus* variety Nagina22 (N22)] and its 137 EMS induced mutants were screened in M4 generation. Of these 137 mutants, 36 mutants were selected based on their grain YLD in low P conditions. Of these 36 mutants, 27 mutants with significantly higher grain YLD than N22 were categorized as hy, and 9 mutants with lower grain YLD or the same YLD as N22 were categorized as ly mutants. These mutants were further characterized for their morpho-agronomical, physiological, and biochemical characteristics.

### Plant Material and Screening

Seeds from the 36 mutants selected in M5 generation were sown. After 25 days, each mutant was transferred to both a low P plot and a normal plot. The low P plot at IIRR was maintained without the application of P fertilizers for 35 years (Olsen P 1.8 kg/ha) (Panigrahy et al., 2014). Nitrogen in the form of urea (100 kg/ha), potassium as a muriate of potash (60 kg/ha), and zinc sulfate (12.5 kg/ha) were applied in both plots as basal dose, except N, which was applied in three split doses. Phosphorus as a single super phosphate (60 kg/ha) was applied in the normal plot only. Spacing was maintained at 20 cm between rows and 15 cm between plants. Soil properties of the experimental plots were as described by Panigrahy et al. (2014). The plants were grown to maturity following the normal package of practices (IRRI SES). Nagina22 (wild type) and Jaya (low P sensitive variety) were included as controls. All morphological, physiological, and biochemical observations were made at the time of 50% flowering.

#### Plant Height (PH)

The PH was measured from the base of the plant to the tip of the main panicle and awns were excluded if present.

#### Tiller Number (TN)

Tiller number for five plants were recorded on a per plant basis.

#### Grain Yield (YLD)

Panicles from each treatment were harvested, threshed, cleaned, and sun dried until the moisture content of seeds dropped to 14% and then weighed. Yield was expressed in g/plant.

#### Chlorophyll a Fluorescence

Chlorophyll a fluorescence parameter, *F*_v_/*F*_m_ and electron transport rate (ETR), were recorded in dark adapted leaves in both treatments using a portable PAM-2100 fluorometer (Walz, Effeltrich, Germany).

#### Phosphorus Concentrations in Roots, Shoots, and Grains

After harvest, the dried roots, shoots, and grains were powdered and digested separately in a mixture of HNO_3_, HClO_4_, and H_2_SO_4_ (3:1:1) at 350°C for 2 h. After digestion, P concentration was determined using a spectrophotometer by the phosphovanadate method (Hanson, 1950).

### Antioxidant Enzymes

#### Superoxide Dismutase (EC 1.15.1.1), Peroxidase (EC 1.11.1.7), and Catalase (EC 1.11.1.6) Activities

Antioxidant enzymes were estimated at tillering (vegetative stage) and flowering (reproductive stage). Enzyme extracts for SOD, POD, and CAT activities were prepared by freezing the weighed amount (0.1 g) of leaf samples at both the vegetative and flowering stages in liquid nitrogen to prevent proteolytic activity, followed by grinding with 5 ml extraction buffer (0.1 M phosphate buffer, pH 7.5, containing 0.5 mM EDTA). The enzyme extract was centrifuged for 20 min at 15000 g at 4C, and the supernatant was collected and assayed for enzyme activity.

The SOD activity was measured according to the method described by Dhindsa et al. (1981). A volume of 3 ml of SOD reaction mixture consisted of methionine (200 mM), nitro blue tetrazolium chloride (NBT) (2.25 mM), EDTA (3.0 mM), riboflavin (60 μM), sodium carbonate (1.5 M), and phosphate buffer (100 mM, pH 7.8). Tubes were kept under light. The reaction mixture without plant extract and irradiation, served as a blank. The absorbance was recorded at 560 nm. The volume of enzyme extract corresponding to 50% inhibition of the reaction was considered as one enzyme unit. Activity was expressed as units/min/g fresh weight (U/min/g FW). Peroxidase assay was carried out according to the method described by Castillo et al. (1984). A volume of 3 ml of POD assay mixture consisted of 1 ml phosphate buffer (pH 6.1), guaiacol 0.5 ml, H_2_O_2_ 0.5 ml, enzyme 0.1 ml, and water 0.9 ml. An increase in absorbance due to the formation of tetraguaiacol was recorded at 470 nm. The POD activity was measured and expressed as μmol of H_2_O_2_ reduced per minute per gram FW. The CAT activity was measured according to the method described by Aebi (1984). The CAT assay mixture of 3 ml consisted of 0.05 ml extract, 1.5 ml phosphate buffer (100 mM buffer, pH 7.0), 0.5 ml H_2_O_2_, and 0.95 ml distilled water. A decrease in the absorbance was recorded at 240 nm. The CAT activity was expressed as μmol of H_2_O_2_ oxidized per minute per gram FW.

### Genotyping

Genomic DNA was extracted from leaves of 36 mutant lines, N22, and Jaya using cetyl trimethyl ammonium bromide extraction buffer. For genotyping, PCR was done with 48 SSR markers (McCouch et al., 2002). The amplified fragments were evaluated as present (1) or absent (0) bands. Single marker analysis was carried out using SMA method in IciMapping v4.1 (www.isbreeding.net) to establish marker trait association.

### Statistical Analysis

Two-way analysis of variance (ANOVA) was carried out using Statistix Ver 8.1. The statistical significance of means for all the parameters was determined by the least significant difference (LSD) test. Principal component analysis (PCA) was done to minimize the dimensions of multivariate data into a few principal axes by generating Eigen vectors for each axis and component scores for the traits using PBTools^1^ (Version 1.4) and R (R Core Team, 2012).

## Results

### Screening of Mutants in Normal and Low P Conditions

For further studies, 27 hy and 9 ly mutants were selected based on grain YLD in low P conditions. These mutants were further screened in normal and low P conditions for detailed physiological and biochemical traits. Grain YLD, TN, and PH at maturity, as well as physiological and biochemical traits at both the vegetative and reproductive stages, were recorded.

#### Grain Yield

**FIGURE 1:**
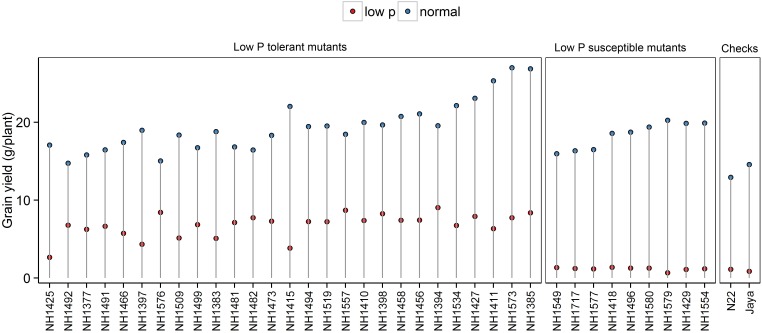
Grain yield (g/plant) in normal and low P conditions in high- and low-yielding mutants.

Significant reduction in grain YLD was observed in low P conditions compared with normal conditions. In low P conditions, there were significant differences among the mutants and controls. Grain YLD ranged from 3 to 9 g/plant in hy mutants and from 0.66 to 1.37 g/plant in ly mutants. When hy mutants were compared with ly mutants, the ly mutants showed 5.74 times less grain YLD under low P conditions. The YLDs of N22 and Jaya were 1.0 and 0.85 g/plant, respectively (Figure 1).

#### Tiller Number

**FIGURE 2:**
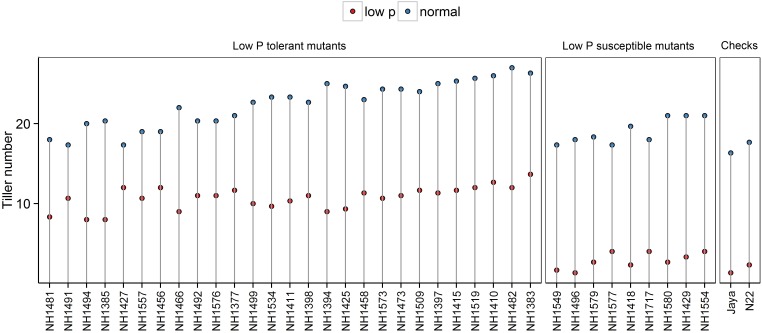
Tiller number in normal and low P conditions in high- and low-yielding mutants.

Tiller numbers were significantly less in low P conditions than in normal conditions. In low P conditions, the differences between hy and ly mutants were significant. In hy mutants, the TN ranged from 8 to 13, and in ly mutants, it ranged from 1 to 4. When hy mutants were compared with ly mutants, the ly mutants showed 73% reduction in TN under low P conditions. The number of tillers per plant in N22 and Jaya were 2 and 1, respectively (Figure 2).

#### Plant Height

**FIGURE 3:**
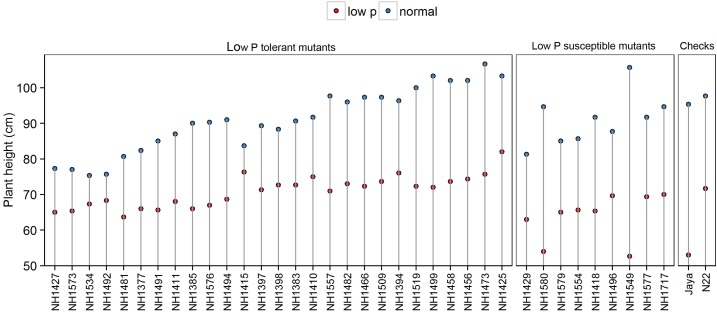
Plant height in normal and low P conditions in high- and low-yielding mutants.

Plant height was 30% less in low P conditions when compared with normal conditions. Significant differences were also observed between the hy and ly mutants. PH ranged from 61 cm to 84 cm in hy mutants and from 50 cm to 72 cm in ly mutants. The ly mutants showed a 10% PH reduction under low P conditions when compared with hy mutants. The PH of N22 and Jaya in low P conditions was 71 cm and 53 cm, respectively (Figure 3).

#### Antioxidant Enzyme Activities

**FIGURE 4:**
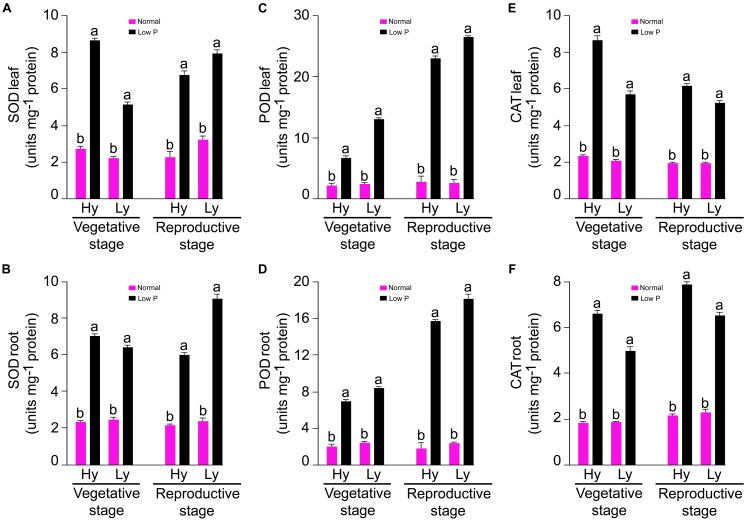
SOD, POD, and CAT activities in leaf **(A,C,E)** and root **(B,D,F)** at vegetative and reproductive stages in high- and low-yielding mutants in normal and low P conditions.

The SOD, POD, and CAT (units mg^-1^ protein) activities were measured in the roots and leaves at both the vegetative (tillering stage) and reproductive stages (panicle initiation) in low P and normal conditions (Supplementary Tables S1, S2). The mean SOD activity in the roots and leaves was threefold more in low P conditions than in normal conditions. At the vegetative stage, differences between mutants were significant. In low P conditions, the average SOD activity in the root and leaves in hy mutants was 9% and 41% higher, respectively, when compared with those in ly mutants (Figure 4A).

At the vegetative stage, differences of the POD activity in the roots and leaves were significant between treatments and mutants. In general, the mean POD activity in the roots and leaves was more than four-fold in low P conditions than in normal conditions. The average root POD activity was 6.87 units mg^-1^ protein in hy mutants and 8.29 units mg^-1^ protein in ly mutants. Thus, in low P conditions, the average root POD activity in ly mutants was 20% higher than hy mutants. In low P conditions, the average leaf POD activity was 12.94 units mg^-1^ protein in hy mutants and 6.52 U/min/g FW in ly mutants (Figure 4C). Thus, in low P conditions, the average leaf POD activity in hy mutants was 98% higher than in ly mutants. At the vegetative stage, differences of the CAT activity in the roots and leaves were significant between treatments and mutants. The mean CAT activity in the roots and leaves of all 36 mutants was more than three-fold in low P conditions than in normal conditions. In hy mutants, the average CAT activity in the roots and leaves was 22% and 35% more, respectively, than ly mutants in low P conditions (Figure 4E).

### SOD Activity

The differences of SOD activity were significant between normal and low P conditions, vegetative and reproductive stages, and hy and ly mutants. In comparison with normal conditions, the mean SOD activity in the roots and leavess was threefold more in low P conditions. The mean SOD activity in the roots and leaves was 0.9% and 18% more, respectively, at the reproductive stage in low P conditions when compared with the vegetative stage. The hy mutants exhibited a 51% and 14% decrease of SOD activity in the roots and leaves during the reproductive stage in low P conditions. On the contrary, the SOD activity in the roots and leaves was 15% and 25% more in ly mutants at the reproductive stage. Nagina22 exhibited a 30% and 80% increase of SOD activity in the roots and leaves during the reproductive stage in low P conditions (Figure 4B).

### POD Activity

Low P conditions triggered significantly more POD activity than normal conditions. The differences of POD activity were significant between the vegetative and reproductive stages and also between hy and ly mutants. In comparison with normal conditions, the mean POD activity in the roots and leaves was ninefold more in low P conditions. The mean POD activity in the roots and leaves was 124% and 185% more at the reproductive stage in low P conditions when compared with the vegetative stage. In low P conditions, the POD activity in the roots and leaves was 14% and 16% higher in ly mutants than in hy mutants. Nagina22 exhibited 115% and 100% more POD activity in the roots and leaves during the reproductive stage in low P conditions (Figure 4D).

### CAT Activity

The differences of CAT activity were significant between low P and normal conditions, vegetative and reproductive stages, as well as between hy and ly mutants. When compared with normal conditions, the mean CAT activity in the roots and leaves was threefold more in low P conditions. The mean CAT activity in the roots and leaves was 22% and 24% more at the reproductive stage in low P conditions than at the vegetative stage. At the reproductive stage, N22 exhibited a 34% and 0.1% increase of CAT activity in the roots and leaves in low P conditions. In comparison with the vegetative stage, CAT activity in ly mutants showed a 15% reduction in roots and a 17% reduction in leaves at the reproductive stage (Figure 4F).

### Chlorophyll a Fluorescence Parameters

**FIGURE 5:**
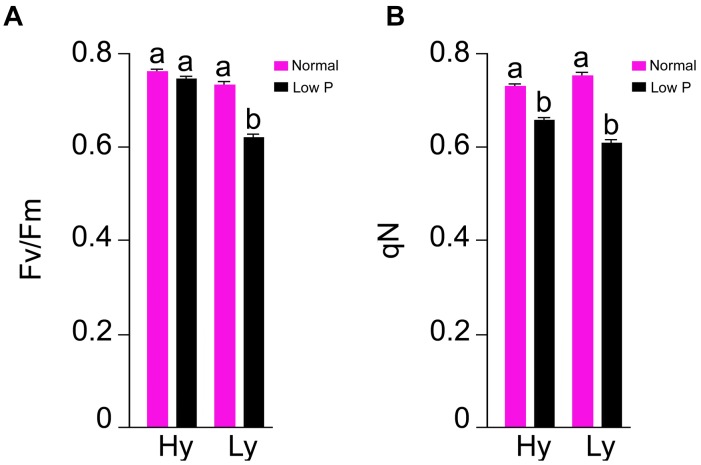
*F*_v_/*F*_m_ **(A)** and q_N_ **(B)** in leaf at reproductive stage in high- and low-yielding mutants in normal and low P conditions.

The parameters *F*_v_/*F*_m_ and q_N_ were measured at the reproductive stage only (Supplementary Table S3). In comparison with normal conditions, there was a 20% reduction in *F*_v_/*F*_m_ and a 15% reduction in q_N_ in low P conditions. The differences of *F*_v_/*F*_m_ and q_N_ were also significant between hy and ly mutants. In low P conditions, the hy mutants showed 16% higher *F*_v_/*F*_m_ and 9% higher q_N_ when compared with ly mutants (Figure 5).

### Phosphorous Concentrations in Roots, Leaves, and Grains

**FIGURE 6:**
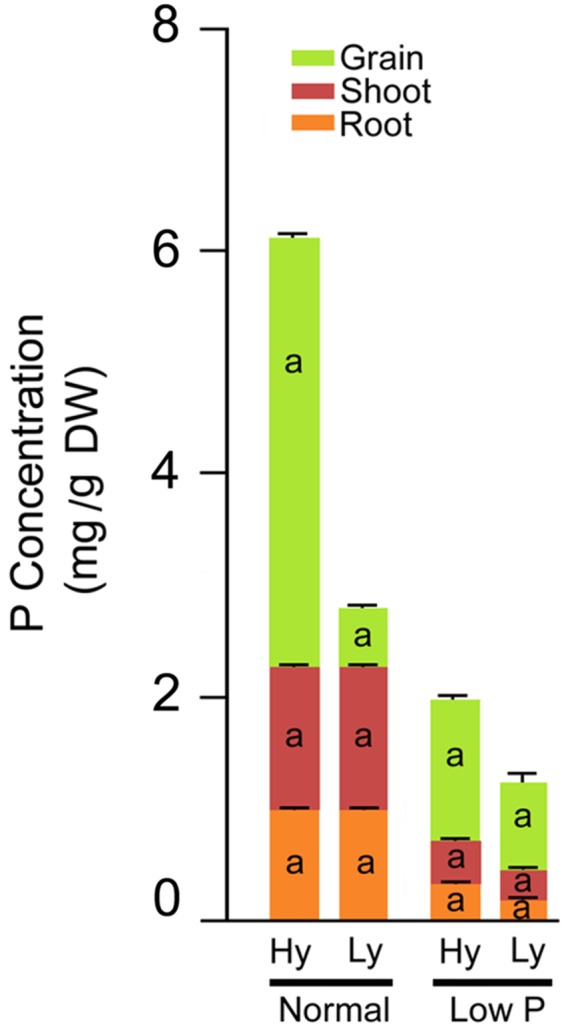
Phosphorus concentration distribution in roots, shoots, and grains in high- and low-yielding mutants in normal and low P conditions.

The differences in total P concentration in roots, leaves, and grains were significant between low and normal P conditions. Significant differences were also noticed among mutants in low P conditions (Supplementary Table S4). The mean root P concentration was significantly higher (63%) in hy mutants when compared with ly mutants. The concentration varied from 0.209 to 2.76 (mg/g DW) in hy mutants and from 0.11 to 0.193 (mg/g DW) in ly mutants. Similarly, in hy mutants, P concentrations were 31% higher in shoots and 39% higher in grains, when compared with those condition in ly mutants in low P conditions. The P concentration range of shoots in hy and ly mutants was 0.309–0.463 (mg/g DW) and 0.213–0.296 (mg/g DW), respectively. Similarly, the P concentration of grains ranged from 1.03 to 1.78 (mg/g DW) in hy mutants and 0.686 to 0.876 (mg/g DW) in ly mutants. Higher P concentration in roots, shoots, and grains eventually led to higher total P concentration in hy mutants. The total P concentration ranged from 1.66 to 4.376 (mg/g DW) in hy mutants and 1.04 to 1.284 (mg/g DW) in ly mutants in low P conditions. Thus, the total P concentration was 43% higher in hy mutants when compared with ly mutants in low P conditions. In N22 and Jaya, the P concentration in roots was 0.23 and 0.17 (mg/g DW), in shoots was 0.34 and 0.26 (mg/g DW), in grains was 0.97 and 0.68 (mg/g DW), and the total P concentration was 1.55 and 1.12 (mg/g DW) respectively, in low P conditions (Figure 6).

### Correlation Coefficients Among SOD, POD, and CAT Activities

**FIGURE 7:**
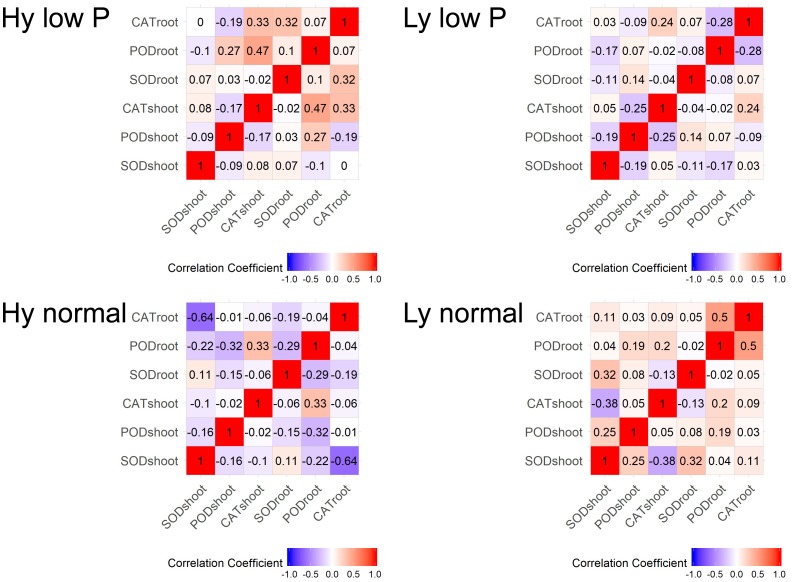
Correlation at vegetative stage in SOD, POD, and CAT activities in high- and low-yielding mutants in normal and low P conditions.

**FIGURE 8:**
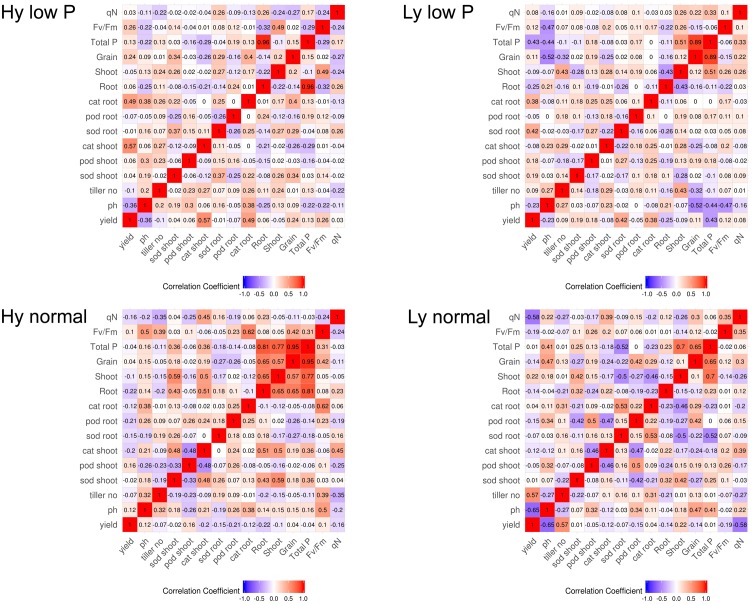
Correlation at reproductive stage in high- and low-yielding mutants in normal and low P conditions.

The correlation among traits at the vegetative stage in hy and ly mutants is shown in Figure 7. In low P conditions at the vegetative stage, the SOD activity in leaves was positively correlated with the CAT activity in both the roots and shoots. The CAT activity in the roots and leaves was negatively correlated with the POD activity in leaves of hy mutants. On the other hand, in ly mutants, a significant positive correlation was observed between the POD activity and the CAT activity in roots. However, the SOD activity was negatively correlated with the CAT activity in roots. In hy mutants, a significant positive correlation was observed between the CAT activity in the roots and shoots, but a negative correlation was observed between the CAT activity in roots, POD activity in roots, and the SOD activity in leaves at normal conditions. In ly mutants at normal conditions, a positive correlation was observed between the POD activity and the CAT activity in roots, but a highly negative correlation was observed between the CAT activity and the SOD activity in shoots (Figure 7). In low P conditions, in hy mutants at the reproductive stage, a positive and significant correlation was found between grain YLD and CAT activity in the roots and shoots, *F*_v_/*F*_m_, and q_N_. In normal conditions, grain YLD was positively correlated with concentrations in P shoots and q_N_. It is important to note that the CAT activity in shoots was positively and significantly correlated with TN in both low P and normal conditions (Figure 8).

### Heat Maps and Clustering in Normal and Low P Conditions

**FIGURE 9:**
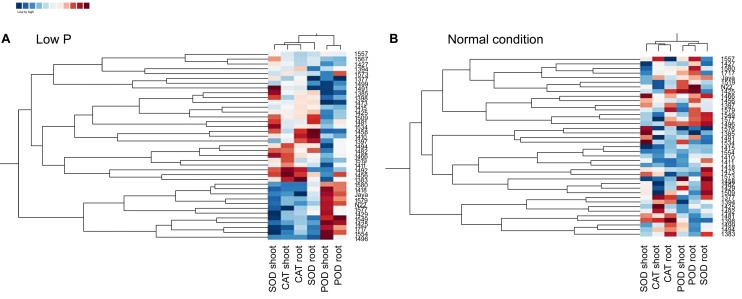
Clustering and correlation at vegetative stage with biochemical assays in selected 36 mutants, N22, and Jaya in normal **(B)** and low P **(A)** conditions.

**FIGURE 10:**
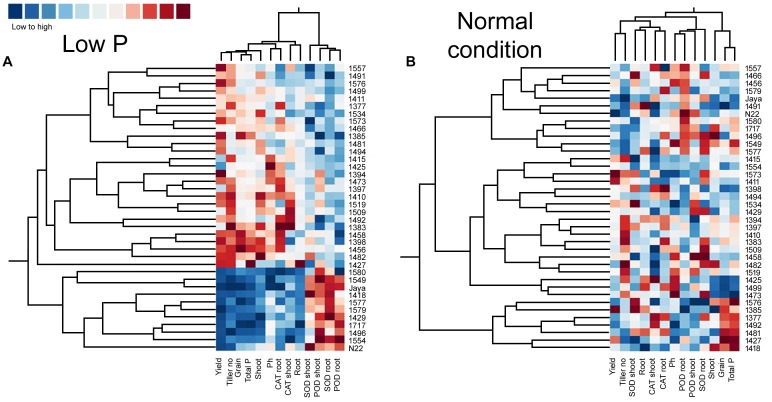
Clustering and correlation at reproductive stage with agronomic traits and biochemical assays in selected 36 mutants, N22, and Jaya in normal **(B)** and low P **(A)** conditions.

Heat maps and clustering of genotypes using different morphological, physiological, and biochemical traits showed a clear grouping of mutants in low P conditions, a specific correlation pattern for all hy mutants, and a reverse pattern for all ly mutants (Figure 9). However, in normal conditions, there was no specific pattern of correlation coefficients between hy and ly mutants for any trait (Figure 10).

### Principal Component Analysis in Normal and Low P Conditions

**FIGURE 11:**
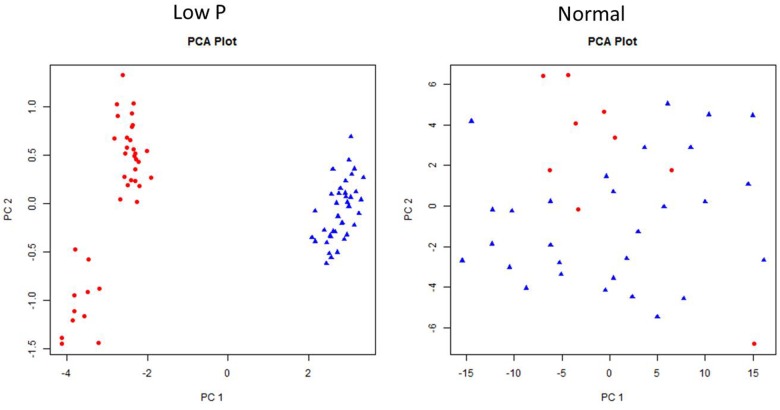
Individual factor map of mutants, N22, and Jaya in low P and normal conditions.

**FIGURE 12:**
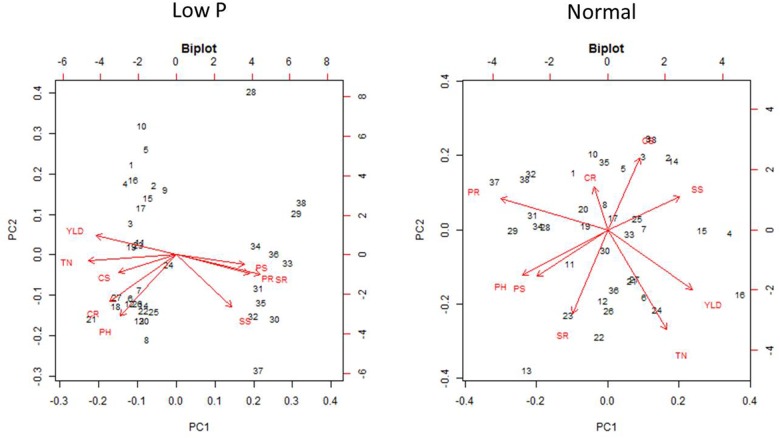
Principal component analysis (PCA) in 36 mutants selected, N22, and Jaya in normal and low P conditions.

Principal component analysis was carried out to establish the relationship between various morphological and biochemical traits under low P and normal conditions (Figure 11). Among the traits, YLD, PH, and TN were detected as main principal components (PCs) differentiating the mutants into various groups. The TN and PH were positive for PC1 and CAT shoots (CS), CAT roots (CR), and POD in roots (PR) were positive for PC2. The first two PCs showed a cumulative proportion of 93% of the variation, under combined analysis. In low P conditions, the PCA showed that SOD shoots (SS), POD shoots (PS), SOD roots (SR), and PR are the key factors/traits that contribute to low P tolerance and differentiate the mutants as tolerant and sensitive. The cumulative proportion of the first two PCs was 69%. The PS, PR, SS, and SR were captured by PC1 and YLD and TN were captured by PC2. The PCA in normal conditions showed that YLD, TN and SS are the key traits that differentiate the mutants into different groups. The cumulative proportion of the first two PCs was only 40%. YLD, TN, and SS were primarily captured by PC1 and CR and PR were captured by PC2. The PCA also showed a random distribution of genotypes in normal conditions, but clustering was observed in low P conditions. In the combined analysis, mutants grown in low P conditions showed clear groupings, but in normal conditions, mutants showed a random distribution (Figure 12).

### Marker Trait Associations

Loci RM3648 and RM451 showed significant association with YLD, TN, SOD activity, and POD activity in both the roots and leaves in only low P conditions, whereas loci RM3334 and RM3600 were associated with only CAT activity in leaves in low P conditions.

## Discussion

Phosphorus deficiency has become a major challenge because of the declining P resources and the increasing cost of P fertilizers, adding a financial burden on farmers of rice growing countries. Phosphorus, a macronutrient, is required for rice cultivation in large amounts. To tackle the problem of P deficiency, identification and development of rice genotypes that can produce higher YLD even in low P soil is a viable approach.

Field testing and screening of 137 mutants, along with N22, was carried out in low P conditions to identify hy mutants that could perform better than N22. Mutants with heat and low P tolerance were identified in field experiments in previous studies (Poli et al., 2013; Panigrahy et al., 2014). Mutants with higher mean YLD/hill than N22 were categorized as hy mutants and those with lower mean YLD/hill than N22 were categorized as ly mutants in low P conditions. The mutants were selected for further screening based on the YLD/hill in low P conditions, and the other traits such as TN/hill and PH were not considered. It was noted from a previous study that although some mutants had a higher TN, they failed to flower; similarly, mutants that had more PH achieved a poor YLD in low P conditions.

Phenotyping of 36 mutants for PH, TN, and YLD/plant showed that hy mutants are more responsive to P and also showed a significant variation between both conditions. The hy mutants showed a range of variations in their phenotypic expression in low P and normal conditions. Across two test environments, NH1576 (TN) and NH1491 and NH1427 (YLD), the TN (Figure 2) and YLD (Figure 3) showed a low range of variation, compared with other mutants. These mutants, showing stable YLD performance across different environments, could be tested under multilocation trials to develop low P tolerant varieties.

Low P conditions trigger increased production of ROS in plants. Antioxidant machinery comprising enzymes (SOD, POD, and CAT) and others are produced as defense molecules to neutralize the ROS, whose uncontrolled production could have a devastating impact at the cellular level on the plant. Most studies on the effect of low P conditions on antioxidant systems were carried out in hydroponics (Xu et al., 2007; You et al., 2014), and there are very few reports of the same in soil conditions (Veronica et al., 2017). Therefore, we investigated the impact of low P soil on the antioxidant enzyme activity in the roots and leaves at both vegetative and reproductive stages of selected hy and ly mutants.

Enhanced activities of antioxidant enzymes (SOD, POD, and CAT) act as a coping strategy to scavenge ROS (Munns and Gilliham, 2015). Overexpression of genes involved in ROS detoxification resulted in lower cellular damage and the maintenance of photosynthetic energy capture under saline conditions (Roy et al., 2014). However, it is unknown whether ROS detoxification occurred because of low P stress. In a previous study, 8-day-old seedlings of an indica cultivar Zhenong 966 were subjected to minus (-) P conditions in hydroponics for 16 days. Both SOD and ascorbate peroxidase (APX) in leaves were significantly higher in -P than in plus (+) P. The parameters *F*_v_/*F*_m_ and q_N_ were also reduced significantly in -P (Xu et al., 2007). In another study, Huanghuazhan, an indica cultivar was used to study the effect of -P conditions in hydroponics for 18 days. Antioxidant enzymes, namely SOD, POD, CAT, APX, and GR, were estimated in leaves, and it was concluded that the SOD and CAT activities were significantly higher in -P when compared with +P conditions (Hussain et al., 2016). Our experiment was designed to decipher the activity of antioxidant enzymes in low P field in both the vegetative and reproductive stages as well as in the roots and leaves. Veronica et al. (2017) screened four rice varieties with different P levels in pot experiments up to maturity: Swarna and Akhanphou were tolerant to low P, and MTU1010 and RPBio 226 were sensitive to low P. Antioxidant enzymes were assayed at the reproductive stage with different P levels. The research team concluded that the SOD, POD, and CAT activities were significantly higher in low P conditions for all the genotypes. In tolerant varieties, the higher CAT activity when accompanied with higher *F*_v_/*F*_m_ resulted in higher grain YLD in low P conditions. In our experiment, we estimated antioxidant enzymes both at the vegetative and reproductive stages in 27 hy and 9 ly mutants of N22 in low P conditions. Antioxidant enzyme activity and photosynthesis showed association with YLD under low P condition but not in normal condition.

In the vegetative stage, elevated SOD and CAT activities in both the roots and leaves of all hy mutants and higher POD activity in ly mutants were observed in low P conditions. Whereas, at the reproductive stage, hy mutants exhibited lower SOD and POD activities but a higher CAT activity in comparison with ly mutants in both the roots and shoots. These results clearly indicate that hy mutants possessed higher SOD and CAT activities at the early stages and a higher CAT activity at the reproductive stage in both the roots and shoots for scavenging the increased ROS levels in low P conditions.

On the other hand, ly mutants had a higher POD activity but lower SOD and CAT activities and were therefore less efficient in scavenging the ROS at the early stages. In ly mutants, a higher POD activity may not be adequate to remove H_2_O_2_ as rapidly as CAT (Alam and Ghosh, 2018). This is likely to result in the accumulation of H_2_O_2_ molecules. It was reported that 10 μM of H_2_O_2_ is sufficient to inhibit 50% of photosynthesis (Kaiser, 1979). Therefore, for the survival of ly mutant plants in low P conditions, a high POD activity alone is insufficient to confer low P tolerance. A higher SOD activity combined with a higher CAT activity at the vegetative stage and a higher CAT activity at the reproductive stage seems to be important for scavenging the increased ROS levels under low P conditions. This may be one of the reasons for better survival and higher YLD of hy mutants under low P conditions.

In the vegetative stage, under low P conditions, in response to the accumulation of ROS, the activity of SOD was increased in both hy and ly mutants that resulted in the accumulation of H_2_O_2_. The hy mutants showed overall lower antioxidant activity only at the reproductive stage when compared with ly mutants. It is important to note that in low P conditions, the SOD activity was higher in hy mutants than in ly mutants at the vegetative stage, and correspondingly the CAT activity was also higher in hy mutants than in ly mutants at the vegetative stage; so, the ROS were effectively scavenged. However, at the critical reproductive stage, CAT activity in ly mutants could not match with the increased SOD activity. Additionally, CAT activity was increasingly efficient in neutralizing H_2_O_2_ when compared with POD activity. The POD activity could keep pace with increased SOD in both hy and ly mutants; so, we concluded that the difference is not due to POD but due to CAT at the reproductive stage. In hy mutants under low P conditions, grain YLD was positively and significantly correlated only with CAT in roots and shoots but not with POD and SOD at the reproductive stage. The hy mutants exhibited higher P concentration in roots, shoots, and grains when compared with ly mutants in low P conditions, suggesting that hy mutants had better P uptake and transport mechanisms that helped the mutants to thrive under low P conditions. This may be one of the reasons for the better survival and YLD of hy mutants under low P conditions.

**FIGURE 13:**
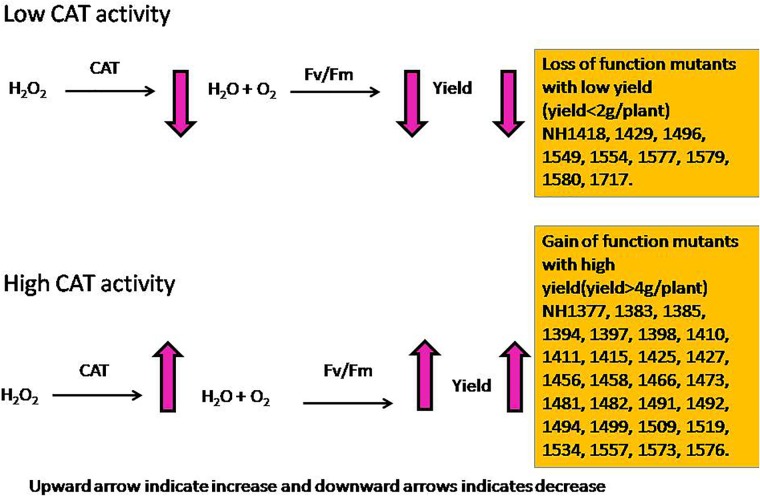
Model diagram of the influence of higher and lower CAT activities on grain yield.

From a physiological point of view, *F*_v_/*F*_m_ that represents the PSII photochemistry was reduced under low P conditions, but it was higher in hy mutants than in ly mutants. The hy mutants also had a higher q_N_ that indicated that excessive excitation energy was lost in the form of heat and minimized the damage caused to photosystem II (Veronica et al., 2017). The hy mutants had higher SOD and CAT activities at the vegetative stage followed by a higher CAT activity at the reproductive stage. Besides, they also had higher *F*_v_/*F*_m_ and q_N_ that indicated that the deleterious effect of low P conditions on PSII mechanism was less in hy mutants. Overall, higher *F*_v_/*F*_m_; qN; and P concentrations in roots, shoots, and grains could probably be the factors promoting hy mutants to cope with the adverse effects of low P conditions. In our study, hy mutants had more CAT activity under low P conditions that resulted in higher *F*_v_/*F*_m_ and q_N_, which favored these mutants for better survival and seed set. On the other hand, in ly mutants, a higher SOD activity at both the stages enhanced H_2_O_2_ concentration. However, CAT activity in ly mutants was less at both stages, which resulted in the accumulation of H_2_O_2_ that inhibited photosynthesis and, thus, reduced grain YLD under low P conditions (Figure 13).

Catalase is the most efficient enzyme, which neutralizes H_2_O_2_ into H_2_O and O_2_ (Mhamdi et al., 2010; Alam et al., 2014; Veronica et al., 2017), and Kcat of CAT was the highest among all the antioxidant enzymes (Singh et al., 2015). This study indicated that low P stress reduces *F*_v_/*F*_m_ in rice, however, mutations in N22 enhanced (in case of hy mutants) or decreased (in case of ly mutants) the activity of CAT, which facilitated the lower reduction of *F*_v_/*F*_m_ in hy mutants as compared with ly mutants. This in turn might be an important factor that led to the higher YLD of hy mutants in low P conditions. It would be interesting to further probe into the effects of antioxidants and ROS pathways in low-P tolerance to establish a link between P metabolism and ROS pathways. It can also facilitate developing a ROS based biomarker to identify the genotypes for low-P tolerance in rice. The N22 mutants analyzed in this study are an important genetic resource for carrying out further analyses in this area of research.

Under normal conditions, the first five components accounted for 99.47% of the total variation and morphological traits like PH, YLD, and TN showing high coefficient values in PC1 (79.1%), indicating their major contribution in discriminating accessions, while the remaining variables had weak or no discriminatory power. In contrast, under low P conditions, PCs up to PC5 exhibited 90.17% variation with TN showing the highest coefficient along with SR, YLD, and PR, explaining the variance. TN is used as a surrogate for measuring tolerance to low-P conditions (Wissuwa et al., 1998; Panigrahy et al., 2014; Poli et al., 2017a). These results, therefore, indicate the impact of antioxidant enzymes in differentiating the genotypes under conditions of stress. Even the pooled analysis has shown that POD and SOD activities in roots contributed more toward differentiating genotypes than the morphological traits with high Eigen values for the first PC and indicated the need to identify and improve genotypes with these factors, for better tolerance under conditions of stress. Multivariate analyses conducted from the biochemical and morphological data of recombinant inbred lines (RIL) from contrasting parents, N22 and IR 64, under drought stress also resolved the significant effect of biochemical factors in differentiating tolerant and sensitive lines, and they found that enzyme-morphology correlations were not significant under irrigated control but were significant in conditions of drought. The researchers concluded that GR had a significant and positive correlation with single plant YLD (SPY) under drought stress but not in normal conditions (Prakash et al., 2016).

Loci RM3648 and RM451 showed significant association with YLD, TN, and SOD and POD activities in both the roots and leaves in low P conditions only. RM3334 and RM3600 were associated with only CAT activity in leaves in low P conditions. The closest genes flanking RM3688 is the oryzain alpha chain precursor (LOC_Os04g55650.1), which is located 5 kb downstream. The gene closest to RM451 is the SNF2 family N-terminal domain containing protein (LOC_Os04g47830.1), which is located 1 kb upstream. RM334 is close to the ankyrin repeat domain-containing protein 28 (LOC_Os05g02130.1), which is located just 124 bp upstream. Close to RM6300 is the mitochondrial import inner membrane translocase subunit Tim17 (LOC_Os05g02060.1), which is located 116 bp downstream. Interestingly, all four genes have a known relationship with antioxidants and are good candidates for establishing a functional link between ROS and low-P tolerance in future studies.

This is the first report to show physiological and biochemical traits together, studying several genotypes (36 mutants + 2 control–N22 and Jaya) in low and normal P conditions, at different growth stages and tissues. Furthermore, the SOD, POD, and CAT activities in the roots and leaves at two stages of crop growth, vegetative and reproductive, as well as the fluorescence parameters, *F*_v_/*F*_m_ and q_N_, at the reproductive stage were analyzed. Higher SOD and CAT activities at the vegetative stage and a higher CAT activity and *F*_v_/*F*_m_ at the reproductive stage are important traits that confer tolerance to low P conditions. This was supported by the high correlation coefficients between SOD and CAT activities at the vegetative stage and CAT activity with grain YLD and *F*_v_/*F*_m_ at the reproductive stage, particularly in low P conditions. We have also shown markers associated (RM3648, RM451, RM3334, and RM3600) with antioxidant enzymes, especially in low P conditions. From this study, the useful chlorophyll fluorescence, antioxidant enzymes, and associated markers were identified, which can be useful for the development of low P tolerant genotypes.

## Author Contributions

SN, SV, SD, SM, and DB designed the research work. YP and VN performed the research. YP, SD, PR, and DB analyzed the data. SN, YP, DB, and SM wrote the manuscript. All authors read and approved the manuscript.

## Conflict of Interest Statement

The authors declare that the research was conducted in the absence of any commercial or financial relationships that could be construed as a potential conflict of interest.

## Footnotes

1 http://bbi.irri.org/products

## References

[B1] Aebi H. (1984). Catalase in vitro. *Meth. Enzymol.* 105 121–126. 10.1016/S0076-6879(84)05016-3 6727660

[B2] Alam N. B. Ghosh A. (2018). Comprehensive analysis and transcript profiling of *Arabidopsis thaliana* and *Oryza sativa* catalase gene family suggests their specific roles in development and stress responses. *Plant Physiol. Biochem.* 123 54–64. 10.1016/j.plaphy.2017.11.018 29223068

[B3] Alam M. M. Nahar K. Hasanuzzaman M. Fujita M. (2014). Trehalose-induced drought stress tolerance: a comparative study among different Brassica species. *Plant Omics J.* 7 271–283. 10.13140/2.1.2883.1366

[B4] Castillo F. I. Penel I. Greppin H. (1984). Peroxidase release induced by ozone in *Sedum album* leaves: involvement of Ca^2+^. *Plant Physiol.* 74 846–851. 10.1104/pp.74.4.846 16663520 10.1104/pp.74.4.846 PMC1066779

[B5] R Core Team (2012). *R: A Language and Environment for Statistical Computing*. Vienna: R Foundation for Statistical Computing.

[B6] Dhindsa R. A. Plumb D. P. Thorpe T. A. (1981). Leaf senescence: correlated with increased permeability and lipid peroxidation, and decreased levels of superoxide dismutase and catalase. *J. Exp. Bot.* 126 93–101. 10.1093/jxb/32.1.93

[B7] Hanson W. C. (1950). The photometric determination of phosphorus in fertilizers using the phosphovanado- molybdate complex. *J. Sci. Food Agric.* 1 172–173. 10.1002/jsfa.2740010604

[B8] Hussain S. Yin H. Peng S. Khan F. A. Khan F. Sameeullah M. (2016). Comparative transcriptional profiling of primed and non primed rice seedlings under submergence stress. *Front. Plant Sci.* 7:1125. 10.3389/fpls.2016.01125 27516766 PMC4964843

[B9] Juszczuk I. M. Wagner A. M. Rychter A. M. (2001). Regulation of alternative oxidase activity during phosphate deficiency in bean roots (*Phaseolus vulgaris*). *Physiol. Plant.* 113 185–192. 10.1034/j.1399-3054.2001.1130205.x 12060295

[B10] Kaiser W. (1979). Carbon metabolism of chloroplasts in the dark. *Planta* 144 193–200. 10.1007/BF00387270 24408693

[B11] Krishnamurthy P. Sreedevi B. Ram T. Padmavathi G. Mahendra K. R. Raghuveer R. P. (2010). Evaluation of rice genotypes for phosphorus use efficiency under soil mineral stress conditions. *Oryza* 47 29–33.

[B12] Lauer M. J. Pallardy S. G. Belvins D. G. Randall D. D. (1989). Whole leaf carbon exchange characteristics of phosphate deficient soybeans (*Glycine max* L.). *Plant Physiol.* 91 848–854. 10.1104/pp.91.3.848 16667147 PMC1062086

[B13] Lima J. M. Nath M. Dokku P. Raman K. V. Kulkarni K. P. Vishwakarma C. (2015). Physiological, anatomical and transcriptional alterations in a rice mutant leading to enhanced water stress tolerance. *AoB Plants* 7:plv023. 10.1093/aobpla/plv023 25818072 PMC4482838

[B14] McCouch S. R. Teytelman L. Xu Y. Lobos K. B. Clare K. Walton M. (2002). Development and mapping of 2240 new SSR markers for rice (*Oryza sativa* L.). *DNA Res.* 9 199–207. 10.1093/dnares/9.6.199 12597276

[B15] Mehra P. Pandey B. K. Giri J. (2017). Improvement in phosphate acquisition and utilization by a secretory purple acid phosphatase (OsPAP21b) in rice. *Plant Biotechnol. J.* 15 1054–1067. 10.1111/pbi.12699 28116829 PMC5506657

[B16] Mhamdi A. Queval G. Chaouch S. Vanderauwera S. Van Breusegem F. Noctor G. (2010). Catalase function in plants: a focus on *Arabidopsis* mutants as stress-mimic models. *J. Exp. Bot.* 61 4197–4220. 10.1093/jxb/erq282 20876333

[B17] Mithra A. S. V. Kar M. K. Mohapatra T. Robin S. Sarla N. Seshashayee M. (2016). DBT propelled national effort in creating mutant resource for functional genomics in rice. *Curr. Sci.* 110 543–548. 10.18520/cs/v110/i4/543-548

[B18] Mohapatra T. Robin S. Sarla N. Sheshashayee M. Singh A. K. Singh K. (2014). EMS induced mutants of upland rice variety nagina22: generation and characterization. *Proc. Indian Natl. Sci. Acad.* 80 163–172. 10.3389/fpls.2018.01179 30233603 PMC6132179

[B19] Munns R. Gilliham M. (2015). Salinity tolerance of crops-what is the cost? *New Phytol.* 208 668–673. 10.1111/nph.13519 26108441

[B20] Pandey B. K. Mehra P. Verma L. Bhadouria J. Giri J. (2017). OsHAD1, a haloacid dehalogenase-like apase, enhances phosphate accumulation. *Plant Physiol.* 174 2316–2332. 10.1104/pp.17.00571 28637831 PMC5543963

[B21] Panigrahy M. Rao D. N. Yugandhar P. Raju S. N. Krishnamurthy P. Voleti S. R. (2014). Hydroponic experiment for identification of tolerance traits developed by rice Nagina 22 mutants to low-phosphorus in field condition. *Arch. Agron. Soil Sci.* 60 565–576. 10.1080/03650340.2013.821197

[B22] Poli Y. Basava R. K. Desiraju S. Voleti S. R. Sharma R. P. Neelamraju S. (2017a). Identifying markers associated with yield traits in Nagina22 rice mutants grown in low phosphorus field or in alternate wet/dry conditions. *Aust. J. Crop Sci.* 11 548–556. 10.1186/1939-8433-6-36 24295086 PMC4883711

[B23] Poli Y. Basava R. K. Panigrahy M. Vinukonda V. P. Dokula N. R. Voleti S. R. (2013). Characterization of a Nagina22 rice mutant for heat tolerance and mapping of yield traits. *Rice* 6:36. 10.1186/1939-8433-6-36 24295086 PMC4883711

[B24] Poli Y. Veronica N. Panigrahy M. Nageswara Rao D. Subrahmanyam D. Voleti S. R. (2017b). Comparing hydroponics, sand, and soil medium to evaluate contrasting rice Nagina 22 mutants for tolerance to phosphorus deficiency. *Crop Sci.* 57 2089–2097.

[B25] Prakash C. Mithra S. V. A. Singh P. K. Mohapatra T. Singh N. K. (2016). Unraveling the molecular basis of oxidative stress management in a drought tolerant rice genotype Nagina 22. *BMC Genomics* 17:774. 10.6084/m9.figshare.c.3624881_d3 27716126 PMC5050613

[B26] Rodríguez D. Keltjens W. G. Goudriaan J. (1998). Plant leaf area expansion and assimilate production in wheat (*Triticum aestivum* L.) growing under low phosphorus conditions. *Plant Soil* 200 227–240. 10.1023/A:1004310217694

[B27] Roy S. J. Negrao S. Tester M. (2014). Salt resistant crop plants. *Curr. Opin. Biotechnol.* 26 115–124. 10.1016/j.copbio.2013.12.004 24679267

[B28] Sanyal S. K. Dwivedi B. S. Singh V. K. Majumdar K. Datta S. C. Pattanayak S. K. (2015). Phosphorus in relation to dominant cropping sequences in India: chemistry, fertility relations and management options. *Curr. Sci.* 108 1262–1270

[B29] Shoba D. Raveendran M. Manonmani S. Utharasu S. Dhivyapriya D. Subhasini G. (2017). Development and genetic characterization of a novel herbicide (imazethapyr) tolerant mutant in rice (*Oryza sativa* L). *Rice* 10:10. 10.1186/s12284-017-0151-8 28378144 PMC5380566

[B30] Singh R. Haukka M. McKenzie C. J. Nordlander E. (2015). High turnover catalase activity of a mixed-valence mn II mn III complex with terminal carboxylate donors. *Eur. J. Inorg. Chem* 2015 3485–3492. 10.1002/ejic.201500468

[B31] Talla S. K. Panigrahy M. Kappara S. Nirosha P. Neelamraju S. Ramanan R. (2016). Cytokinin delays dark-induced senescence in rice by maintaining the chlorophyll cycle and photosynthetic complexes. *J. Exp. Bot.* 67 1839–1851. 10.1093/jxb/erv575 26826216 PMC4783366

[B32] Vandamme E. Wissuwa M. Rose T. Dieng I. Drame K. N. Fofana M. (2016). Genotypic variation in grain P loading across diverse rice growing environments and implications for field P balances. *Front. Plant Sci.* 7:1435. 10.3389/fpls.2016.01435 27729916 PMC5037189

[B33] Veronica N. Subrahmanyam D. Kiran T. V. Yugandhar P. Bhadana V. P. Padma V. (2017). Influence of low phosphorus concentration on leaf photosynthetic characteristics and antioxidant response of rice genotypes. *Photosynthetica* 55 285–293. 10.1007/s11099-016-0640-4

[B34] Wissuwa M. Yano M. Ae N. (1998). Mapping of QTLs for phosphorus-deficiency tolerance in rice (*Oryza sativa* L.). *Theor. Appl. Genet.* 97 777–783. 10.1007/s001220050955

[B35] Xu X. H. Weng X. Y. Yang Y. (2007). Effect of phosphorus deficiency on the photosynthetic characteristics of rice plants. *Russ. J. Plant Physiol.* 54 741–748. 10.1134/S1021443707060040

[B36] You L. Wood S. U. Fritz S. Guo Z. See L. Koo J. (2014). *Spatial Production Allocation Model (SPAM) 2005 v2.0*. Available at: http://mapspam.info

[B37] Yugandhar P. Nallamothu V. Panigrahy M. Sitaramamma T. Bhadana V. P. Voleti S. R. (2018b). Nagina 22 mutants tolerant or sensitive to low P in field show contrasting response to double P in hydroponics and pots. *Arch. Agron. Soil Sci.* 28:17Z 10.1080/03650340.2018.1471684

[B38] Yugandhar P. Yafei Sun Y. Liu L. Negi M. Nallamothu N. Sun S. (2018a). Characterization of the loss-of-function mutant NH101 for yield under phosphate deficiency from EMS-induced mutants of rice variety Nagina22. *Plant Physiol. Biochem.* 130 1–13. 10.1016/j.plaphy.2018.06.017 29957570

