# Supplementary material for: Increased Catalase Activity and Maintenance of Photosystem II Distinguishes High-Yield Mutants From Low-Yield Mutants of Rice var. Nagina22 Under Low-Phosphorus Stress
Source: Front Plant Sci. 2018 Nov 19;9:1543. doi: 10.3389/fpls.2018.01543 (PMC6252357; doi:10.3389/fpls.2018.01543)
Supplement: Supplementary file 1 [file Table_1.DOC]

Supplementary table 1. Antioxidant enzyme activities in 36 mutants at vegetative stage in low P and normal condition

| Mutants | SOD (Shoot) | | SOD (Root) | | POD (Shoot) | | POD (Root) | | CAT (Shoot) | | CAT (Root) | |
| --- | --- | --- | --- | --- | --- | --- | --- | --- | --- | --- | --- | --- |
|  | Low P | Normal | Low P | Normal | Low P | Normal | Low P | Normal | Low P | Normal | Low P | Normal |
| NH1557 | 6.26 | 2.26 | 5.24 | 1.42 | 9.50 | 2.50 | 7.33 | 2.73 | 4.23 | 3.02 | 5.41 | 1.07 |
| NH1576 | 7.14 | 2.96 | 5.33 | 1.14 | 7.33 | 1.42 | 6.47 | 1.49 | 7.49 | 1.73 | 5.56 | 1.67 |
| NH1377 | 6.69 | 2.18 | 4.29 | 2.22 | 6.38 | 1.70 | 5.22 | 1.78 | 6.67 | 3.19 | 6.26 | 2.30 |
| NH1385 | 8.18 | 3.06 | 5.89 | 1.56 | 5.43 | 1.85 | 5.60 | 1.27 | 8.01 | 2.26 | 6.55 | 1.70 |
| NH1427 | 6.62 | 1.17 | 5.32 | 1.33 | 6.60 | 1.96 | 6.54 | 2.38 | 7.89 | 2.29 | 5.60 | 1.70 |
| NH1415 | 5.74 | 1.13 | 5.66 | 1.61 | 7.37 | 2.15 | 6.44 | 1.81 | 8.22 | 1.81 | 6.70 | 1.56 |
| NH1394 | 6.11 | 1.97 | 4.62 | 1.94 | 7.89 | 1.45 | 7.55 | 1.92 | 7.40 | 2.70 | 6.85 | 2.22 |
| NH1425 | 6.50 | 1.78 | 5.92 | 1.67 | 6.41 | 1.77 | 6.67 | 2.23 | 8.52 | 3.34 | 6.63 | 1.63 |
| NH1481 | 7.55 | 2.56 | 6.32 | 1.72 | 7.04 | 2.07 | 7.85 | 1.60 | 7.44 | 2.54 | 6.05 | 2.26 |
| NH1491 | 8.52 | 2.57 | 4.33 | 1.36 | 5.45 | 2.09 | 6.35 | 2.48 | 7.55 | 1.70 | 5.90 | 1.59 |
| NH1499 | 5.40 | 1.58 | 4.78 | 1.88 | 5.43 | 2.59 | 6.04 | 1.63 | 8.19 | 2.18 | 6.45 | 2.26 |
| NH1473 | 6.44 | 2.32 | 5.84 | 2.37 | 5.22 | 2.01 | 5.40 | 1.82 | 7.67 | 2.30 | 6.63 | 1.70 |
| NH1458 | 7.44 | 1.21 | 6.78 | 2.45 | 5.94 | 3.27 | 5.74 | 2.20 | 7.03 | 1.81 | 7.43 | 1.54 |
| NH1398 | 7.53 | 2.66 | 5.71 | 1.33 | 5.97 | 2.39 | 6.38 | 1.51 | 6.74 | 2.96 | 6.67 | 2.58 |
| NH1534 | 8.23 | 3.11 | 5.81 | 1.33 | 7.40 | 2.85 | 7.43 | 1.63 | 7.44 | 2.12 | 6.50 | 1.55 |
| NH1573 | 5.67 | 2.63 | 5.22 | 2.24 | 6.37 | 1.85 | 8.59 | 1.59 | 8.81 | 2.59 | 5.88 | 1.16 |
| NH1494 | 6.70 | 1.76 | 5.76 | 1.89 | 5.48 | 2.55 | 7.53 | 1.59 | 10.30 | 2.63 | 6.63 | 2.19 |
| NH1492 | 7.41 | 2.07 | 4.70 | 1.88 | 6.37 | 1.77 | 6.63 | 1.78 | 11.71 | 3.08 | 7.66 | 1.78 |
| NH1466 | 8.11 | 3.04 | 5.59 | 2.27 | 7.19 | 2.33 | 7.45 | 2.46 | 10.31 | 2.35 | 6.68 | 2.15 |
| NH1456 | 7.53 | 2.48 | 5.89 | 1.84 | 6.62 | 2.05 | 8.66 | 2.57 | 12.14 | 2.39 | 7.40 | 1.78 |
| NH1383 | 6.70 | 1.83 | 5.49 | 2.23 | 5.74 | 2.07 | 7.58 | 1.70 | 10.89 | 2.26 | 8.21 | 2.40 |
| NH1482 | 7.39 | 2.23 | 6.18 | 2.30 | 6.21 | 2.52 | 7.52 | 1.82 | 9.98 | 1.70 | 6.37 | 1.63 |
| NH1519 | 6.67 | 1.66 | 5.46 | 1.70 | 6.82 | 2.82 | 6.56 | 2.29 | 10.26 | 1.78 | 5.63 | 1.66 |
| NH1411 | 6.06 | 2.11 | 5.26 | 2.17 | 5.59 | 1.80 | 7.45 | 2.20 | 9.81 | 1.85 | 6.51 | 1.28 |
| NH1397 | 4.99 | 2.56 | 6.47 | 1.81 | 6.49 | 1.74 | 7.62 | 2.34 | 7.63 | 2.20 | 7.30 | 2.23 |
| NH1509 | 7.37 | 2.29 | 6.29 | 2.38 | 6.29 | 2.34 | 6.69 | 1.59 | 8.55 | 1.70 | 6.70 | 2.13 |
| NH1410 | 6.67 | 1.81 | 6.74 | 1.81 | 7.70 | 1.76 | 6.32 | 2.06 | 8.08 | 1.93 | 7.66 | 1.74 |
| NH1580 | 4.74 | 1.32 | 5.34 | 1.81 | 11.17 | 2.47 | 8.45 | 2.72 | 5.30 | 2.37 | 4.48 | 1.99 |
| NH1549 | 3.70 | 1.80 | 4.59 | 2.29 | 12.28 | 1.96 | 9.56 | 2.56 | 6.48 | 1.84 | 5.34 | 1.85 |
| NH1418 | 4.11 | 1.56 | 5.62 | 2.03 | 13.55 | 1.78 | 8.45 | 1.89 | 5.34 | 2.00 | 4.51 | 1.29 |
| NH1577 | 4.07 | 1.81 | 5.41 | 2.26 | 12.82 | 2.22 | 7.66 | 2.54 | 7.45 | 1.70 | 5.52 | 2.15 |
| NH1717 | 3.73 | 1.47 | 4.63 | 1.40 | 12.83 | 2.65 | 8.30 | 2.56 | 4.78 | 2.26 | 5.61 | 1.92 |
| NH1554 | 3.63 | 1.43 | 4.70 | 1.60 | 13.96 | 1.84 | 8.59 | 1.85 | 5.41 | 1.89 | 5.40 | 1.59 |
| NH1496 | 4.55 | 1.75 | 4.48 | 2.34 | 14.15 | 2.77 | 7.66 | 2.59 | 5.70 | 2.12 | 4.74 | 2.26 |
| NH1579 | 4.13 | 2.04 | 5.26 | 1.80 | 12.70 | 1.63 | 8.36 | 2.56 | 6.34 | 2.07 | 5.14 | 2.42 |
| NH1429 | 3.64 | 2.26 | 5.55 | 2.30 | 13.07 | 2.95 | 7.62 | 1.78 | 6.44 | 1.78 | 5.52 | 1.66 |
| N22 | 4.21 | 2.21 | 5.08 | 1.59 | 12.89 | 2.82 | 8.63 | 2.92 | 6.33 | 1.78 | 4.77 | 1.70 |
| Jaya | 4.45 | 1.66 | 5.30 | 2.15 | 13.23 | 2.47 | 8.59 | 2.43 | 5.77 | 2.70 | 3.78 | 1.74 |
| T(LSD<0.05) | 0.0635 |  | 0.0713 |  | 0.0594 |  | 0.0716 |  | 0.0587 |  | 0.0662 |  |
| M(LSD<0.05) | 0.277 |  | 0.3108 |  | 0.2587 |  | 0.3122 |  | 0.2557 |  | 0.2887 |  |
| TXM(LSD<0.05) | 0.3917 |  | 0.4395 |  | 0.3659 |  | 0.4415 |  | 0.3616 |  | 0.4083 |  |
