# Supplementary material for: Increased Catalase Activity and Maintenance of Photosystem II Distinguishes High-Yield Mutants From Low-Yield Mutants of Rice var. Nagina22 Under Low-Phosphorus Stress
Source: Front Plant Sci. 2018 Nov 19;9:1543. doi: 10.3389/fpls.2018.01543 (PMC6252357; doi:10.3389/fpls.2018.01543)
Supplement: Supplementary file 2 [file Table_2.DOC]

Supplementary table 2. Antioxidant enzyme activities in 36 mutants at reproductive stage in low P and normal condition

| Mutants | SOD (Shoot) | | SOD (Root) | | POD (Shoot) | | POD (Root) | | CAT (Shoot) | | CAT (Root) | |
| --- | --- | --- | --- | --- | --- | --- | --- | --- | --- | --- | --- | --- |
|  | Low P | Normal | Low P | Normal | Low P | Normal | Low P | Normal | Low P | Normal | Low P | Normal |
| NH1557 | 4.27 | 1.39 | 4.42 | 2.10 | 22.52 | 3.37 | 16.59 | 1.44 | 6.59 | 1.32 | 6.43 | 1.59 |
| NH1576 | 5.40 | 1.10 | 4.59 | 1.39 | 23.70 | 3.23 | 15.81 | 2.25 | 5.48 | 2.40 | 6.88 | 2.14 |
| NH1377 | 4.98 | 1.56 | 4.44 | 1.42 | 20.05 | 2.67 | 16.59 | 1.41 | 5.59 | 1.54 | 8.59 | 1.64 |
| NH1385 | 5.36 | 2.35 | 4.52 | 1.91 | 18.94 | 3.07 | 14.59 | 1.55 | 6.06 | 1.44 | 7.59 | 2.58 |
| NH1427 | 4.53 | 1.59 | 5.09 | 2.46 | 25.39 | 2.85 | 15.19 | 1.44 | 6.47 | 1.42 | 6.61 | 2.62 |
| NH1415 | 5.31 | 1.54 | 4.59 | 1.96 | 22.52 | 3.26 | 15.35 | 1.44 | 6.45 | 1.89 | 7.87 | 2.34 |
| NH1394 | 5.56 | 1.16 | 4.74 | 2.20 | 25.81 | 2.77 | 15.26 | 1.54 | 5.74 | 1.52 | 8.17 | 1.55 |
| NH1425 | 5.40 | 2.49 | 4.70 | 1.44 | 22.54 | 2.62 | 15.80 | 1.40 | 5.41 | 1.59 | 8.19 | 2.62 |
| NH1481 | 5.74 | 2.20 | 5.12 | 1.26 | 22.51 | 2.66 | 15.56 | 2.28 | 6.29 | 1.61 | 6.78 | 1.54 |
| NH1491 | 4.55 | 1.46 | 3.38 | 1.32 | 22.70 | 2.52 | 15.72 | 1.59 | 5.54 | 2.14 | 6.59 | 2.22 |
| NH1499 | 5.42 | 1.93 | 4.29 | 1.76 | 22.63 | 1.98 | 15.57 | 2.37 | 5.63 | 1.65 | 7.66 | 1.48 |
| NH1473 | 5.15 | 1.39 | 4.44 | 1.59 | 25.43 | 2.77 | 16.26 | 1.51 | 6.31 | 2.67 | 8.52 | 2.48 |
| NH1458 | 6.02 | 1.56 | 5.57 | 1.59 | 24.66 | 2.93 | 14.52 | 1.59 | 5.57 | 2.20 | 8.78 | 2.59 |
| NH1398 | 6.32 | 1.33 | 5.52 | 2.56 | 22.72 | 2.78 | 14.84 | 1.45 | 4.82 | 1.58 | 8.66 | 1.59 |
| NH1534 | 5.33 | 2.64 | 4.86 | 1.46 | 20.70 | 2.97 | 15.32 | 1.45 | 5.12 | 1.59 | 7.56 | 2.41 |
| NH1573 | 4.19 | 1.52 | 4.52 | 2.24 | 23.41 | 2.77 | 16.28 | 2.74 | 5.78 | 1.55 | 8.33 | 2.78 |
| NH1494 | 5.30 | 2.53 | 5.32 | 1.87 | 19.44 | 2.84 | 15.41 | 2.31 | 6.44 | 2.49 | 7.12 | 2.39 |
| NH1492 | 6.25 | 1.40 | 4.44 | 1.54 | 19.26 | 2.52 | 16.04 | 1.48 | 7.39 | 1.65 | 8.58 | 2.47 |
| NH1466 | 4.59 | 1.59 | 4.51 | 1.44 | 22.77 | 2.43 | 16.04 | 1.59 | 6.45 | 1.59 | 8.15 | 1.38 |
| NH1456 | 5.63 | 1.75 | 5.26 | 1.82 | 24.66 | 2.67 | 16.78 | 1.64 | 5.78 | 1.53 | 8.59 | 2.15 |
| NH1383 | 5.14 | 2.49 | 5.14 | 1.44 | 19.96 | 2.07 | 14.88 | 2.18 | 7.70 | 1.46 | 9.16 | 1.62 |
| NH1482 | 6.68 | 1.56 | 5.14 | 1.43 | 22.38 | 2.82 | 15.56 | 2.28 | 6.15 | 2.41 | 7.57 | 2.56 |
| NH1519 | 5.43 | 2.34 | 4.65 | 2.34 | 25.44 | 2.94 | 15.30 | 1.51 | 7.08 | 2.51 | 6.76 | 2.22 |
| NH1411 | 6.23 | 1.91 | 4.52 | 1.63 | 24.47 | 2.51 | 16.46 | 1.37 | 5.45 | 2.76 | 7.65 | 2.41 |
| NH1397 | 5.33 | 1.58 | 5.43 | 1.48 | 25.56 | 2.21 | 16.30 | 1.59 | 6.36 | 2.60 | 8.64 | 1.48 |
| NH1509 | 5.36 | 1.59 | 5.44 | 1.48 | 24.55 | 2.95 | 15.53 | 2.22 | 7.43 | 2.49 | 7.77 | 2.47 |
| NH1410 | 5.19 | 2.29 | 4.76 | 1.34 | 23.77 | 2.24 | 15.70 | 2.35 | 6.74 | 2.22 | 8.33 | 2.49 |
| NH1580 | 5.25 | 2.25 | 6.39 | 2.18 | 26.56 | 2.83 | 14.38 | 3.29 | 4.66 | 1.22 | 5.89 | 2.68 |
| NH1549 | 6.59 | 2.78 | 7.45 | 1.59 | 27.96 | 2.55 | 18.47 | 2.18 | 5.20 | 1.85 | 6.59 | 2.61 |
| NH1418 | 7.59 | 2.56 | 7.78 | 1.59 | 26.21 | 2.76 | 16.60 | 2.45 | 4.85 | 1.59 | 6.78 | 2.53 |
| NH1577 | 6.36 | 2.82 | 7.54 | 1.49 | 25.77 | 2.56 | 17.59 | 2.36 | 5.34 | 1.98 | 6.20 | 1.70 |
| NH1717 | 6.26 | 2.64 | 6.56 | 1.61 | 25.90 | 2.33 | 19.41 | 1.81 | 5.37 | 2.34 | 6.93 | 1.46 |
| NH1554 | 5.74 | 2.57 | 7.63 | 2.40 | 27.97 | 2.55 | 20.22 | 1.73 | 4.52 | 2.19 | 6.45 | 2.55 |
| NH1496 | 5.35 | 2.77 | 5.99 | 1.44 | 27.54 | 2.27 | 18.40 | 1.74 | 4.79 | 1.59 | 6.58 | 1.56 |
| NH1579 | 6.41 | 2.97 | 7.63 | 2.27 | 25.28 | 2.19 | 17.93 | 1.47 | 5.27 | 2.17 | 7.36 | 2.56 |
| NH1429 | 5.79 | 2.11 | 8.30 | 1.59 | 26.55 | 1.74 | 18.52 | 2.42 | 5.52 | 2.17 | 6.52 | 2.51 |
| N22 | 7.57 | 2.48 | 6.59 | 2.37 | 25.81 | 1.84 | 18.58 | 1.96 | 6.34 | 2.24 | 6.39 | 2.65 |
| Jaya | 6.62 | 2.34 | 7.59 | 2.43 | 26.19 | 3.15 | 19.11 | 3.41 | 5.56 | 1.60 | 5.86 | 2.52 |
| T(LSD<0.05) | 0.0751 |  | 0.0711 |  | 0.1236 |  | 0.0892 |  | 0.07 |  | 0.0789 |  |
| M(LSD<0.05) | 0.3273 |  | 0.3101 |  | 0.5386 |  | 0.3886 |  | 0.3052 |  | 0.344 |  |
| TXM(LSD<0.05) | 0.4629 |  | 0.4385 |  | 0.7617 |  | 0.5496 |  | 0.4317 |  | 0.4865 |  |
