# Supplementary material for: Increased Catalase Activity and Maintenance of Photosystem II Distinguishes High-Yield Mutants From Low-Yield Mutants of Rice var. Nagina22 Under Low-Phosphorus Stress
Source: Front Plant Sci. 2018 Nov 19;9:1543. doi: 10.3389/fpls.2018.01543 (PMC6252357; doi:10.3389/fpls.2018.01543)
Supplement: Supplementary file 3 [file Table_3.DOC]

Supplementary table 3. P concentration in root, shoot, grain and total in 36 mutants after harvesting in low P and normal condition

| Mutants | P concentration (Root) | | P concentration (Shoot) | | P concentration (Grain) | | P concentration (Total) | |
| --- | --- | --- | --- | --- | --- | --- | --- | --- |
|  | Low P | Normal | Low P | Normal | Low P | Normal | Low P | Normal |
| NH1557 | 0.22 | 0.98 | 0.32 | 1.22 | 1.21 | 4.09 | 1.75 | 6.28 |
| NH1576 | 0.23 | 0.98 | 0.35 | 1.55 | 1.10 | 4.27 | 1.68 | 6.81 |
| NH1377 | 0.27 | 0.93 | 0.35 | 1.30 | 1.27 | 4.31 | 1.89 | 6.55 |
| NH1385 | 0.31 | 1.12 | 0.34 | 0.88 | 1.57 | 4.35 | 2.21 | 6.35 |
| NH1427 | 0.31 | 0.88 | 0.32 | 1.24 | 1.14 | 4.66 | 1.77 | 6.78 |
| NH1415 | 0.29 | 0.96 | 0.36 | 1.31 | 1.14 | 3.78 | 1.78 | 6.05 |
| NH1394 | 0.30 | 1.12 | 0.33 | 1.48 | 1.30 | 3.78 | 1.92 | 6.38 |
| NH1425 | 0.32 | 1.13 | 0.35 | 0.99 | 1.18 | 3.34 | 1.85 | 5.45 |
| NH1481 | 0.25 | 1.14 | 0.39 | 0.98 | 1.26 | 4.49 | 1.91 | 6.61 |
| NH1491 | 0.24 | 1.27 | 0.40 | 0.97 | 1.16 | 3.28 | 1.80 | 5.52 |
| NH1499 | 0.29 | 1.17 | 0.39 | 0.98 | 1.08 | 3.33 | 1.76 | 5.47 |
| NH1473 | 0.26 | 0.97 | 0.37 | 0.96 | 1.20 | 3.34 | 1.83 | 5.27 |
| NH1458 | 0.21 | 0.93 | 0.38 | 1.19 | 1.57 | 3.64 | 2.16 | 5.77 |
| NH1398 | 0.32 | 0.86 | 0.42 | 1.25 | 1.53 | 3.39 | 2.26 | 5.50 |
| NH1534 | 0.22 | 0.89 | 0.42 | 1.15 | 1.32 | 3.78 | 1.95 | 5.82 |
| NH1573 | 0.28 | 0.85 | 0.38 | 1.44 | 1.26 | 3.86 | 1.93 | 6.14 |
| NH1494 | 0.29 | 0.92 | 0.39 | 1.15 | 1.08 | 3.70 | 1.76 | 5.77 |
| NH1492 | 0.29 | 0.95 | 0.32 | 1.33 | 1.22 | 4.23 | 1.82 | 6.52 |
| NH1466 | 0.34 | 0.94 | 0.38 | 1.23 | 1.30 | 3.57 | 2.01 | 5.74 |
| NH1456 | 0.31 | 0.92 | 0.41 | 1.22 | 1.66 | 3.77 | 2.39 | 5.92 |
| NH1383 | 0.32 | 0.94 | 0.42 | 1.18 | 1.36 | 4.13 | 2.10 | 6.24 |
| NH1482 | 0.28 | 0.94 | 0.45 | 1.74 | 1.34 | 3.66 | 2.07 | 6.34 |
| NH1519 | 0.27 | 0.98 | 0.40 | 1.48 | 1.19 | 3.73 | 1.87 | 6.20 |
| NH1411 | 0.29 | 0.97 | 0.37 | 1.24 | 1.32 | 3.67 | 1.98 | 5.87 |
| NH1397 | 0.33 | 0.98 | 0.38 | 1.45 | 1.23 | 3.87 | 1.94 | 6.30 |
| NH1509 | 0.31 | 0.95 | 0.39 | 1.34 | 1.14 | 3.76 | 1.85 | 6.06 |
| NH1410 | 0.36 | 0.96 | 0.44 | 1.44 | 1.24 | 3.87 | 2.03 | 6.27 |
| NH1580 | 0.15 | 0.96 | 0.26 | 1.34 | 0.87 | 4.07 | 1.28 | 6.38 |
| NH1549 | 0.17 | 0.99 | 0.28 | 1.56 | 0.75 | 4.05 | 1.20 | 6.60 |
| NH1418 | 0.17 | 0.75 | 0.26 | 1.74 | 0.78 | 4.27 | 1.21 | 6.76 |
| NH1577 | 0.17 | 1.15 | 0.29 | 1.36 | 0.69 | 3.88 | 1.14 | 6.38 |
| NH1717 | 0.12 | 0.97 | 0.24 | 1.65 | 0.69 | 3.74 | 1.05 | 6.36 |
| NH1554 | 0.18 | 0.98 | 0.26 | 1.35 | 0.82 | 3.45 | 1.26 | 5.78 |
| NH1496 | 0.17 | 0.98 | 0.25 | 1.86 | 0.75 | 3.44 | 1.16 | 6.28 |
| NH1579 | 0.18 | 0.96 | 0.27 | 1.28 | 0.79 | 3.56 | 1.24 | 5.80 |
| NH1429 | 0.19 | 0.98 | 0.22 | 0.98 | 0.76 | 3.85 | 1.17 | 5.82 |
| N22 | 0.24 | 1.09 | 0.34 | 1.08 | 0.97 | 3.86 | 1.55 | 6.02 |
| Jaya | 0.18 | 0.85 | 0.27 | 0.99 | 0.68 | 3.65 | 1.13 | 5.48 |
| T(LSD<0.05) | 0.0182 |  | 0.0164 |  | 0.0222 |  | 0.0321 |  |
| M(LSD<0.05) | 0.0793 |  | 0.0713 |  | 0.0966 |  | 0.1401 |  |
| TXM(LSD<0.05) | 0.1122 |  | 0.1008 |  | 0.1366 |  | 0.1981 |  |
