# Supplementary material for: Increased Catalase Activity and Maintenance of Photosystem II Distinguishes High-Yield Mutants From Low-Yield Mutants of Rice var. Nagina22 Under Low-Phosphorus Stress
Source: Front Plant Sci. 2018 Nov 19;9:1543. doi: 10.3389/fpls.2018.01543 (PMC6252357; doi:10.3389/fpls.2018.01543)
Supplement: Supplementary file 4 [file Table_4.DOC]

Supplementary table 4. Fv/Fm and qN in 36 mutants after harvesting in low P and normal conditions.

| Mutants | Fv/Fm | | qN | |
| --- | --- | --- | --- | --- |
|  | Low P | Normal | Low P | Normal |
| NH1557 | 0.707 | 0.717 | 0.822 | 0.923 |
| NH1576 | 0.706 | 0.721 | 0.82 | 0.924 |
| NH1377 | 0.707 | 0.719 | 0.819 | 0.933 |
| NH1385 | 0.707 | 0.729 | 0.952 | 0.989 |
| NH1427 | 0.706 | 0.731 | 0.952 | 0.991 |
| NH1415 | 0.705 | 0.732 | 0.85 | 0.999 |
| NH1394 | 0.707 | 0.737 | 0.842 | 0.932 |
| NH1425 | 0.703 | 0.739 | 0.845 | 0.921 |
| NH1481 | 0.704 | 0.736 | 0.85 | 0.945 |
| NH1491 | 0.707 | 0.733 | 0.845 | 0.901 |
| NH1499 | 0.706 | 0.735 | 0.85 | 0.913 |
| NH1473 | 0.705 | 0.739 | 0.849 | 0.914 |
| NH1458 | 0.707 | 0.747 | 0.846 | 0.923 |
| NH1398 | 0.708 | 0.746 | 0.857 | 0.922 |
| NH1534 | 0.707 | 0.749 | 0.863 | 0.924 |
| NH1573 | 0.707 | 0.767 | 0.817 | 0.923 |
| NH1494 | 0.708 | 0.768 | 0.822 | 0.912 |
| NH1492 | 0.709 | 0.769 | 0.815 | 0.923 |
| NH1466 | 0.707 | 0.745 | 0.902 | 0.966 |
| NH1456 | 0.709 | 0.747 | 0.902 | 0.978 |
| NH1383 | 0.708 | 0.749 | 0.902 | 0.954 |
| NH1482 | 0.707 | 0.733 | 0.822 | 0.961 |
| NH1519 | 0.704 | 0.736 | 0.826 | 0.928 |
| NH1411 | 0.705 | 0.735 | 0.828 | 0.901 |
| NH1397 | 0.752 | 0.767 | 0.876 | 0.908 |
| NH1509 | 0.753 | 0.766 | 0.879 | 0.91 |
| NH1410 | 0.754 | 0.769 | 0.886 | 0.923 |
| NH1580 | 0.603 | 0.713 | 0.746 | 0.911 |
| NH1549 | 0.604 | 0.716 | 0.746 | 0.912 |
| NH1418 | 0.605 | 0.717 | 0.751 | 0.921 |
| NH1577 | 0.626 | 0.723 | 0.822 | 0.923 |
| NH1717 | 0.627 | 0.725 | 0.827 | 0.925 |
| NH1554 | 0.628 | 0.726 | 0.829 | 0.935 |
| NH1496 | 0.621 | 0.744 | 0.706 | 0.945 |
| NH1579 | 0.623 | 0.742 | 0.71 | 0.956 |
| NH1429 | 0.622 | 0.746 | 0.712 | 0.955 |
| N22 | 0.594 | 0.754 | 0.766 | 0.978 |
| Jaya | 0.595 | 0.756 | 0.765 | 0.985 |
| T(LSD<0.05) | 0.0121 |  | 0.0156 |  |
| M(LSD<0.05) | 0.0342 |  | 0.0412 |  |
| TXM(LSD<0.05) | 0.1243 |  | 0.1094 |  |
